# Data-Driven Optimization of Discontinuous and Continuous Fiber Composite Processes Using Machine Learning: A Review

**DOI:** 10.3390/polym17182557

**Published:** 2025-09-22

**Authors:** Ivan Malashin, Dmitry Martysyuk, Vadim Tynchenko, Andrei Gantimurov, Vladimir Nelyub, Aleksei Borodulin

**Affiliations:** Artificial Intelligence Technology Scientific and Education Center, Bauman Moscow State Technical University, 105005 Moscow, Russia

**Keywords:** machine learning, fiber-reinforced composites, graph neural networks, pultrusion, reinforcement learning

## Abstract

This paper surveys the application of machine learning in fiber composite manufacturing, highlighting its role in adaptive process control, defect detection, and real-time quality assurance. First, the need for ML in composite processing is highlighted, followed by a review of data-driven approaches—including predictive modeling, sensor fusion, and adaptive control—that address material heterogeneity and process variability. An in-depth analysis examines six case studies, among which are XPBD-based surrogates for RL-driven robotic draping, hyperspectral imaging (HSI) with U-Net segmentation for adhesion prediction, and CNN-driven surrogate optimization for variable-geometry forming. Building on these insights, a hybrid AI model architecture is proposed for natural-fiber composites, integrating a physics-informed GNN surrogate, a 3D Spectral-UNet for defect segmentation, and a cross-attention controller for closed-loop parameter adjustment. Validation on synthetic data—including visualizations of HSI segmentation, graph topologies, and controller action weights—demonstrates end-to-end operability. The discussion addresses interpretability, domain randomization, and sim-to-real transfer and highlights emerging trends such as physics-informed neural networks and digital twins. This paper concludes by outlining future challenges in small-data regimes and industrial scalability, thereby providing a comprehensive roadmap for ML-enabled composite manufacturing.

## 1. Introduction

Composite materials, renowned for their high strength-to-weight ratio and design flexibility, have become indispensable in aerospace, automotive, and construction industries. However, the manufacturing processes for composites are often complex, involving multi-physics phenomena and stringent quality requirements. Traditional trial-and-error approaches and physics-based simulations can be time-consuming and computationally expensive, limiting the scalability of production. Enter machine learning (ML)—a paradigm that leverages data-driven models to optimize, predict, and control manufacturing processes with unprecedented speed and accuracy.

This review explores ML applications in both discontinuous and continuous fiber composite manufacturing processes, such as chopped fiber spraying and pultrusion—two distinct paradigms that nonetheless share common challenges in process monitoring, quality control, and optimization. Spray-up process is a method for producing complex-shaped composite parts by depositing resin-impregnated chopped fibers onto a mold, followed by compaction with a roller. Despite its flexibility, challenges such as achieving uniform fiber distribution, minimizing wrinkles, and optimizing robotic paths for complex geometries remain. Pultrusion, on the other hand, is a continuous process for manufacturing constant-cross-section profiles. It involves pulling fiber reinforcements through a resin bath and a heated die for curing. Key challenges include real-time monitoring of process parameters (e.g., temperature, pull speed), ensuring consistent curing, and predicting mechanical properties of the final product. The former enables complex geometries but requires adaptive control of stochastic material deposition; the latter demands precision in continuous, multi-physical production. Both generate data-rich environments where traditional modeling falls short, making them ideal candidates for ML-driven innovation.

The integration of ML into these processes has emerged as a transformative solution. For instance, in spray-up process, ML techniques like reinforcement learning (RL) with physics-based surrogates have enabled robotic draping optimization, while hyperspectral imaging (HSI) combined with deep learning has improved adhesion prediction for metallic coatings. In pultrusion, ML-driven frameworks facilitate synchronized data acquisition, prediction of fracture toughness from standard tests, and estimation of structural performance from geometric parameters.

To ensure reproducibility, a structured methodology for the literature selection was applied. Publications were identified through searches in Scopus using combinations of keywords such as “machine learning,” “deep learning,” “surrogate modeling,” “fiber-reinforced composites,” “pultrusion,” “resin transfer molding,” “injection molding,” “automated fiber placement,” and “structural optimization.” The search period covered 2010–2025, reflecting the sharp rise in ML adoption during this time. Inclusion criteria required that the studies apply ML methods directly to composite processes or performance prediction, with either experimental validation, numerical benchmarks, or open dataset release. Exclusion criteria eliminated purely papers not in English, those limited to generic polymer systems without reinforcement, and papers describing these processes without ML context.

**Figure 1:**
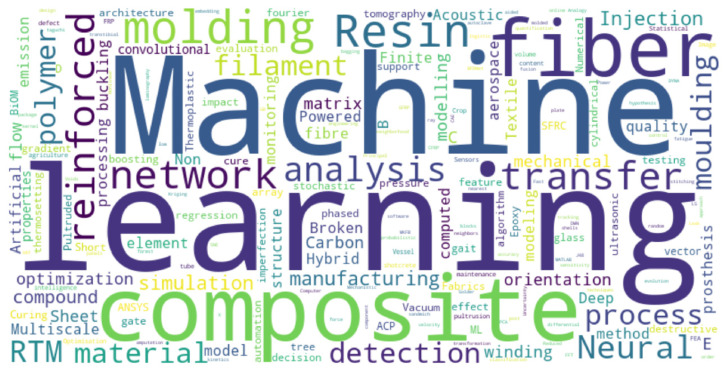
Word cloud of frequently used terms in publications related to ML applications in composite manufacturing.

To identify key themes in the intersection of ML and fiber-reinforced composite manufacturing, a word cloud was generated from the relevant literature and is shown in Figure 1 (based on Scopus data). The visualization highlights commonly occurring terms in publications related to ML applications in composite manufacturing. Prominent words include “machine,” “learning,” “composite,” “fiber,” “network,” and “molding,” reflecting widespread research interest in predictive modeling, process simulation, and fiber-reinforced materials. The size of each word corresponds to its frequency across the literature corpus.

**Figure 2:**
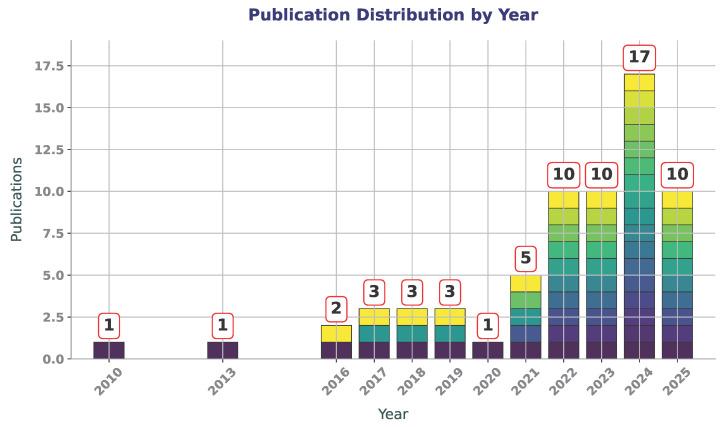
Annual distribution of publications on ML in composite manufacturing from 2010 to 2025.

To track the temporal development of research on ML applications in composite manufacturing, the number of related publications was analyzed by year and is shown in Figure 2. The bar plot provides an overview of how interest in this topic has evolved over time. A notable increase is observed from 2021 onward, with a peak in 2024, indicating growing research activity and attention to the integration of ML techniques in this field.

**Figure 3:**
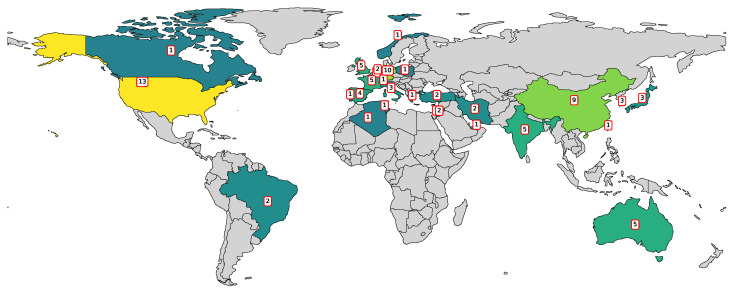
Geographic distribution of publications related to ML in composite manufacturing.

To understand the global distribution of research activity on ML applications in composite manufacturing, publication data were analyzed by country. The resulting map in Figure 3 illustrates the number of contributions from each country, indicating geographical trends in this area of study. The United States, Germany, and China lead in the number of contributions, followed by countries such as Australia, France, India, and the United Kingdom. The map reflects a broad international interest, with research efforts spanning across North America, Europe, Asia, and other regions.

Despite rapid progress in integrating ML into fiber-reinforced composite technologies, a comprehensive synthesis that spans the full range of manufacturing methods and material systems remains scarce. This review addresses that gap by:

- Systematically surveying recent advances in ML applications across diverse composite manufacturing processes—including Automated Composite Fiber Placement (ACFP), pultrusion, Resin Transfer Molding (RTM), Sheet Molding Compounds (SMC), and additive and spray-based techniques.
- Highlighting emerging trends, cross-cutting challenges, and open research questions at the intersection of ML, materials engineering, and process control.
- Proposing and demonstrating a hybrid physics-informed ML model tailored for predicting and optimizing composite process outcomes under limited data regimes.
- Emphasizing the role of hybrid physics–ML models, real-time monitoring, digital twins, and surrogate modeling in advancing performance, efficiency, and automation in composite manufacturing.

The remainder of this paper is organized as follows. Section 2 highlights the need for ML in composite manufacturing, motivating the integration of data-driven strategies into fiber composite fabrication processes. Section 3 presents an overview of data-driven approaches in composite materials, including predictive modeling, sensor fusion, and adaptive process control. Section 4 provides an in-depth research analysis, highlighting recent advances, ongoing challenges, and key gaps in the application of ML to composite manufacturing. Section 5 details the proposed AI model architecture for natural fiber-based composite fabrication, combining spectral segmentation (SpectralUNet), graph neural networks, and a cross-attention controller for adaptive decision-making. Section 6 delivers a broader discussion on issues such as interpretability, scalability, and industrial deployment. Finally, Section 7 presents the conclusion and outlines directions for future research toward intelligent and sustainable composite manufacturing systems.

## 2. Need for ML in Composite Manufacturing

### 2.1. Data-Driven Innovations in Polymer and Composite Materials

The intersection of polymer science and ML has expanded the scope of rational material design, enabling breakthroughs across synthesis, performance prediction, and sustainability. In polymer synthesis, Cao et al. [1] demonstrated closed-loop ML frameworks that integrate polymerization kinetics with structural control, thereby enabling autonomous strategies that directly link molecular architectures to bulk properties. In the context of thermal property optimization, Althagafi et al. [2] developed a machine-assisted pipeline to identify polymers with tailored thermal conductivity, where Random Forest models screened over 10,000 candidates and prioritized synthesizable, high-performance formulations.

Applications in FRP structural engineering further illustrate the benefits of ML-driven modeling. Yu et al. [3], Qu et al. [4], and Zhang et al. [5] applied methods such as SVM, ANN, and GAN–ANN hybrids to predict debonding strain, interfacial bond strength, and stability coefficients, respectively. These approaches addressed the challenges of nonlinearity, limited experimental datasets, and interpretability, yielding predictive tools of practical relevance to civil engineering design. Complementary advances have also been made in polymer-based energy and electronic systems. Wang et al. reviewed third-component engineering in organic solar cells, highlighting opportunities for ML in donor–acceptor optimization, while Kabiru et al. [6]. outlined the role of coordination polymers in supercapacitors, with ML emerging as a pathway for predicting redox activity and facilitating green synthesis. Similarly, Zhang et al. [7] surveyed flexible thermally conductive composites, emphasizing machine learning-assisted strategies to optimize thermal conductivity, flexibility, and process scalability.

Parallel progress has been achieved in smart and adaptive polymers. Kennedy et al. [8] discussed ML-enhanced self-healing composites for biomedical applications, Liao et al. [9] reported bioinspired adhesive hydrogels designed through protein database mining and ML-guided optimization, and Cadamuro et al. [10] proposed predictive platforms for ECM-mimetic hydrogels, improving reproducibility in regenerative medicine. In epoxy systems, Chen et al. [11] synthesized advances in multiscale rheological characterization and identified ML-assisted formulation design as a promising route toward multifunctional and sustainable resin matrices.

A transformative direction has been the emergence of inverse and generative design. Hassan et al. [12] employed graph neural networks and transformer-assisted generative models for bandgap-tunable polymers, while Li et al. [9] integrated natural language processing-based sequence representations with GAN-augmented data to predict glass transition temperatures. Parvez and Mehedi [13] developed the SMILES-PPDCPOA deep learning framework, achieving high accuracy in polymer property classification across diverse datasets. In parallel, Zheng et al. [14] demonstrated the educational translation of these methods by introducing simulation-driven formulation and ML-supported problem solving into polymer engineering curricula, highlighting the role of predictive tools in training the next generation of engineers.

Collectively, these contributions demonstrate how ML enhances polymer engineering by accelerating discovery, improving interpretability of structure–property relationships, bridging experiments with computation, and enabling sustainable and scalable solutions in energy, biomedical, and structural applications. This synergy establishes ML-assisted polymer design as a transformative paradigm that moves beyond trial-and-error experimentation toward predictive, data-driven, and eco-conscious innovation.

### 2.2. Data-Driven Solutions for Composite Manufacturing

Discontinuous and continuous fiber processes raise varied technical hurdles that directly inform ML design and deployment. Discontinuous processes, such as injection molding or automated chopped fiber spraying, are characterized by stochastic fiber orientation, heterogeneous microstructures, and high variability in mechanical properties [15]. These features make accurate prediction and process optimization difficult using conventional models, but ML methods like surrogate modeling [16], image-based learning [17], and reinforcement learning [18] have shown promise in capturing complex variability and enabling adaptive control.

By contrast, continuous processes such as automated fiber placement, pultrusion, or resin transfer molding are typically more structured but require precise control of process parameters [19], defect detection in real time, and integration with digital twins for scalability. Here, ML approaches such as convolutional networks [20], graph neural networks [21], and hybrid physics-informed surrogates [22] are effective in monitoring, detecting anomalies, and guiding optimization under strict quality and throughput constraints.

**Figure 4:**
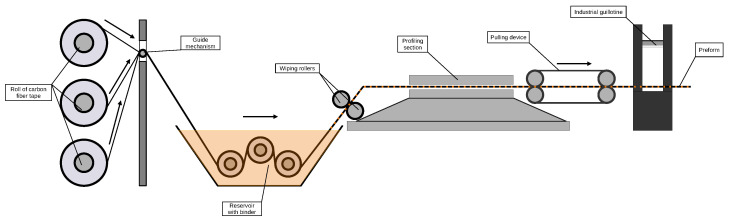
Schematic diagram of the pultrusion process.

Pultrusion, a process developed in the mid-20th century, combines pulling and extrusion to create composite profiles but faces several challenges [23,24,25]. Key process challenges include controlling the pulling speed and die temperature, which impact product quality and can lead to poor performance or failure if mismanaged [26]. Material-wise, thermoset resins are brittle and emit volatile compounds, necessitating careful handling, whereas thermoplastics, though advantageous in toughness and recyclability, suffer from high melt viscosity, causing poor fiber impregnation. Additionally, handling glass fibers presents health risks, driving interest in natural alternatives. The industry also faces challenges in accurately modeling the process to optimize product properties and continues to seek improvements in manufacturing capabilities, heating behavior, and pulling speed control. Environmental and economic considerations further complicate the selection of sustainable and cost-effective materials. A schematic diagram of the tape-laying process in pultrusion is shown in Figure 4.

**Figure 5:**
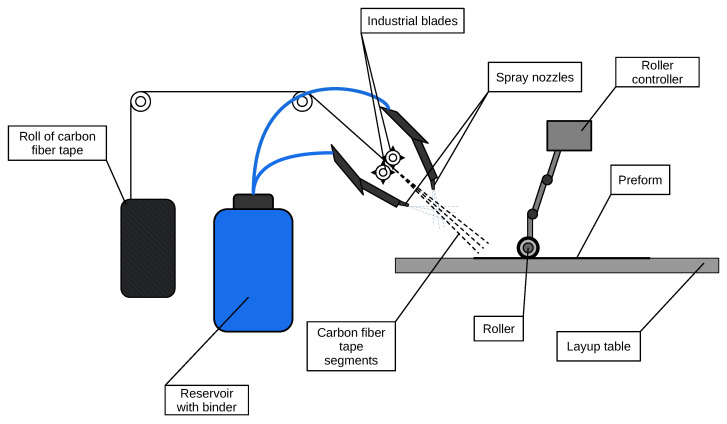
Automated chopped fiber spraying.

Spray-up process is a process in which carbon fiber tape is fed through industrial blades that cut it into segments (Figure 5). These segments then pass through jets of sprayed resin and are deposited onto a layup table. On the table, a specialized roller shapes and compresses the material, forming either simple plates or complex-shaped products. The roller can be either manual or automated, as shown in the image. This method allows for the efficient production of composite materials with high precision and repeatability. The use of an automated process enhances productivity and ensures consistent quality, making it ideal for mass production and creating products with required mechanical properties and geometry [27,28,29].

In composite manufacturing, several challenges underscore the need for ML techniques. One major issue is the process variability due to differences in material properties, environmental conditions, and equipment performance [30,31]. ML can develop robust control strategies that adapt to these variations, ensuring consistent product quality. Quality assurance and defect reduction [32] influence manufacturing, where maintaining high standards and minimizing defects impact yield and waste reduction. Continuous monitoring of manufacturing processes to detect anomalies and predict maintenance needs helps keep production lines running smoothly and efficiently. ML aids in real-time defect detection and prediction [33].

Process optimization is an ongoing challenge in manufacturing. ML models can analyze historical data to identify optimal process settings, predict outcomes, and reduce operational costs while maintaining quality [34,35]. Material characterization and property prediction ensure product performance. ML accelerates material characterization by predicting properties from simpler tests, reducing reliance on extensive and costly experimental campaigns [36,37,38].

Manufacturing also involves integrating multimodal data from various sources such as sensors, imaging systems, and process logs [39]. Data fusion is a key factor in monitoring and control within composite manufacturing processes [40]. Additionally, custom and complex geometries demand flexible manufacturing processes capable of adapting to varied design specifications. ML models assist in managing such variability for consistent quality across different designs [41,42].

**Figure 6:**
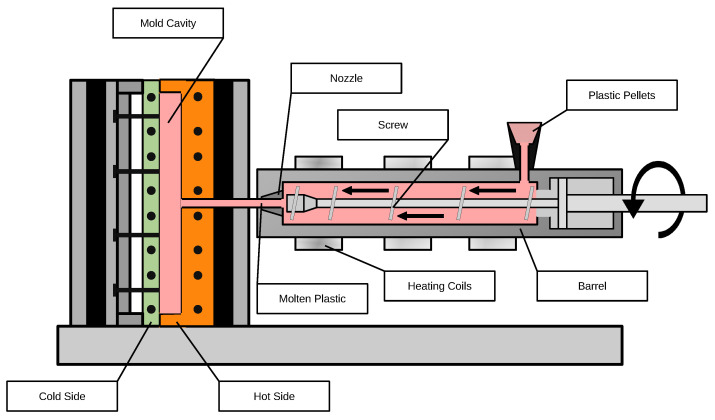
Injection molding of short-fiber composites.

Injection molding is a manufacturing process used to produce short fiber-reinforced composite parts by injecting a mixture of polymer melt and chopped fibers into a mold cavity under high pressure (Figure 6). The process begins with the blending of thermoplastic pellets and short fibers, which are then fed into the injection unit. Inside the barrel, the material is melted and thoroughly mixed by a rotating screw before being injected into the mold. The mold is designed to form the desired geometry and surface quality of the part. Once the material cools and solidifies, the mold opens, and the finished part is ejected. This method enables high-volume production of complex, lightweight components with enhanced mechanical properties, making it suitable for applications in the automotive, aerospace, and consumer goods industries [43,44,45].

Table 1 provides a comparison of composite manufacturing processes, analyzing their key challenges, advantages, ML integration opportunities, and underlying scientific principles. This integration of ML addresses fundamental limitations: computational bottlenecks in physics simulation, sparse experimental data for complex properties, and variability in geometry/material inputs. Cross-cutting needs include explainable models preserving physical constraints (e.g., energy conservation in XPBD) and robust uncertainty quantification for safety-critical applications.

## 3. Data-Driven Approaches in Composite Materials

The recent literature on ML-enabled methods for fiber-reinforced composites and related materials spans a wide array of applications—from interfacial wetting to real-time defect detection and process-parameter optimization. In studies of porous fibrous interfaces, Ma et al. [102] combined high-speed imaging, image-recognition-driven geometry extraction, and finite-element simulations to predict droplet spreading and infiltration on dried tobacco leaf surfaces, revealing how microstructure governs competing wetting and evaporation phenomena. In the realm of continuous-fiber production, Li et al. [103] developed BFDNet, a RetinaNet-based network with multiscale kernel fusion, coupled to a novel strand-numbering algorithm for in-line detection and tracking of broken carbon fibers during winding.

Work on material selection and process design has been advanced by frameworks that integrate physics simulation with Bayesian optimization and neural networks. Yao et al. [104] built a multi-fidelity Bayesian optimization loop around a finite-element creep-strain model of solder joints, automatically identifying epoxy molding compounds that minimized fatigue damage under thermal cycling. Dos Santos and Castro compared support-vector machines, kriging, and random forests as surrogate drivers in an imperfection-sensitive cylinder design, demonstrating ML-guided trade-off frontiers between mass and stiffness when applying continuous tow shearing. Perin et al. [105] similarly paired automated finite-volume simulations with a gradient-boosting-based gate-location predictor to optimize fiber-orientation distributions in injection-molded thermoplastics, achieving over 90% prediction accuracy and experimentally validating local stiffness gains.

Imaging and sensor-based monitoring approaches were also prominent. Castro et al. used in situ synchrotron-computed laminography and a trained segmentation network to track resin-transfer impregnation fronts and void evolution in woven fabric preforms, while Azizian and Almeida [106] employed Taguchi screening and boosted-tree regression to quantify manufacturing uncertainty in filament-wound cylinders. Esmaeili et al. [107] introduced an electrical-circuit analogue for vacuum-bag leak detection, generating synthetic flow-rate data to train ML classifiers that located leaks with over 94% accuracy. In addition, Caliskan [108] applied single-layer neural networks to predict static and impact properties of sheet-molding-compound panels, and Dhanesh et al. [109] combined MATLAB-based image analysis with polynomial regression to forecast low-velocity impact energy absorption in stitched foam-filled honeycomb sandwiches.

Several studies tackled advanced modeling and classification tasks across domains. Ren et al. [110] presented “Machine Eye for Defects,” integrating object detection, vision transformers, and segment-anything models to locate and characterize topological defects in nematic textures. Wei et al. [111] embedded a deep-material network within LS-DYNA to model nonlinear anisotropy of injection-molded short-fiber composites at multiscale speeds orders of magnitude faster than direct simulation. Lemoyne and Mastroianni [112] trained decision trees, k-NN, logistic regression, and SVMs on gait-analysis features to classify prosthesis control architectures, achieving 95% accuracy. Yun et al. [113] used manifold embedding (t-SNE, PCA) to map fabric heterogeneity parameters in VARTM, enabling rapid prediction of void-formation variability from permeability statistics. Collectively, these works illustrate evolving trends toward hybrid physics--ML frameworks, in-line vision and sensor integration, and data-driven process and material design across composite manufacturing and related fields.

Recent years have seen a surge of research at the intersection of ML and automated process monitoring in fiber-reinforced composites. Tunukovic et al. [114] introduced a human–machine collaborative framework for ultrasonic phased-array inspection of carbon-fiber-reinforced plastics, exploring three levels of AI integration—from suggestion-driven assistance to fully autonomous, multi-model analysis pipelines. Applied to complex aerospace components, their system combined supervised object detection on C-scans, unsupervised anomaly detection on B-scans, and a self-supervised volumetric model, yielding a 17.2% boost in F1 score and reducing analysis time from hours to under two minutes. Alshannaq and Awawdeh [115,116] leveraged gradient boosting on the extensive literature data to predict pin-bearing strength of aged and non-aged pultruded GFRP, revealing systematic biases in existing design codes and proposing revised formulae to improve safety and cost-effectiveness in structural applications.

In the realm of process modeling, Wang et al. employed an image-based PixelRNN metamodel to emulate resin flow in resin-transfer molding (RTM), achieving 97.3% accuracy with half the computational cost of physics-based simulations. Chai et al. [117] complemented this by releasing a comprehensive dataset of RTM filling patterns, establishing a benchmark for future data-driven models. Lee and Sohn [118] generated 1369 FEA simulations of filament--wound composite shells under external loads and compared multiple regression and tree-based ML methods, identifying random forests and XGBoost as top performers for buckling-pressure prediction in marine and aerospace engineering.

Several contributions focused on in situ imaging and sensor integration. Mendoza et al. [119] paired high-resolution X-ray tomography with unsupervised ML to automate strain-pattern classification in woven composites, while Wu et al. [120] trained support-vector and genetic-algorithm-optimized neural networks to predict thermoset curing curves from simulated temperature–time histories. Lizarralde et al. [121] used ML-driven segmentation of XCT volumes to quantify micro-crack initiation in non-crimp fabric laminates under thermal cycling, and Esmaeili et al. [107] devised an electrical-circuit analogue to generate synthetic vacuum-bag leak data, training classifiers that located defects with over 94% accuracy and slashed diagnostic time from minutes to seconds.

Optimization and uncertainty quantification studies have also benefited from ML. Pfrommer et al. [122] applied deep-network surrogates to reduce the number of costly finite-element simulations in textile draping, and Azizian and Almeida [106] combined Taguchi screening with boosted-tree regression to assess the reliability of filament--wound cylinders under pressure. Dos Santos and Castro [123] compared SVM, kriging, and random-forest surrogates to optimize continuous-tow-sheared cylinder designs, delineating mass-stiffness trade-off frontiers. Finally, Karamov et al. [124] demonstrated that gradient-boosted decision trees trained on standard mechanical test data could predict fracture toughness of pultruded composites within experimental error, highlighting ML’s potential to bridge macro- and micro-scale material design. These studies chart a trajectory toward hybrid physics--ML frameworks, real-time defect detection, and data-driven design optimization across composite manufacturing.

The integration of ML with numerical simulation has begun to transform traditional composite manufacturing workflows by providing rapid, data-driven predictions of process outcomes. In Sheet Molding Compound (SMC) production, Tannous et al. [125] developed a “digital twin” that couples Darcy-Law reverse-flow simulations with an ML model trained on mold geometry and charge thickness parameters to predict the optimal initial preform shape. This approach matched the accuracy of full simulations while reducing computation time and avoiding convergence failures, and it lays the groundwork for extending charge-shape optimization to complex three-dimensional molds.

Beyond SMC, Berenstein et al. [126] demonstrated the value of ML-enhanced vision in an agricultural composite context: four computer-vision algorithms—combining statistical measures, learned features, and shape matching—were deployed on vineyard imagery to distinguish grape clusters from foliage. This selective-spraying system achieved 90% accuracy in cluster detection and reduced pesticide use by 30%, illustrating how ML can support precision processes in environments with high noise and variability.

In the domain of shotcrete and sprayed composites, Alkan et al. [127] applied an AdaBoost-based neural network to predict material rebound in dry-mix shotcrete applications. Drawing on experimental data from fly-ash and polypropylene-fiber-reinforced mixes, the model outperformed conventional ML algorithms on the same dataset—achieving 84.3% accuracy—and identified mix constituents that minimize rebound loss. These studies reveal a common theme: hybrid frameworks that integrate physics-based modeling, high-fidelity data generation, and tailored ML architectures can deliver rapid, reliable predictions that reduce trial and error, conserve materials, and streamline both industrial and field-scale composite processes.

Recent efforts to integrate ML with composite manufacturing have focused on closing the loop between simulation, in-line sensing, and real-time decision making. Appels et al. [128] investigated fiber-volume-fraction (FVF) gradients in cryogenic hydrogen tanks manufactured by filament winding. By embedding piezoresistive sensors during cure and applying a convolutional-segmentation network to micrographs, they quantified through-thickness FVF variations of up to 11.6%pt. in one resin system, correlating these gradients to mandrel expansion and resin flow. Their results offered actionable guidelines for selecting resin formulations and cure cycles that minimize FVF heterogeneity in Type V liquid-hydrogen vessels.

In high-speed molding processes, stochastic variability of material properties and flow behaviors can undermine finite-element predictions. Iqbal and Xiao [129] addressed this in sheet-molding-compound (SMC) crashworthiness simulations by replacing classical unimodal strength distributions with bimodal Weibull models whose parameters were tuned via an artificial-neural-network-driven optimization loop. When applied to three- and four-point bending cases, their probabilistic ML-augmented FE framework converged within 20–30 iterations and reduced prediction errors across all loading modes.

Real-time process monitoring has also benefited from digital-twin architectures. Stieber et al. [130] embedded multiple sensor modalities in a thermoplastic RTM mold and combined their outputs with an ML-powered digital twin to track polymerization progress of ε-caprolactam. This framework not only visualized flow fronts and degree of cure but also forecasted deviations and suggested corrective actions before defects could form.

Optimizing flow media layouts by reinforcement learning has proven effective for vacuum-assisted processes. Szarski and Chauhan [131] trained a deep-RL agent on 3D FE simulations of VARTM infusion to place flow channels in thin aerospace laminates, achieving a 32% reduction in fill time on complex geometries while avoiding resin starvation.

Accurate prediction of fiber orientation and resulting mechanical properties underpins structural performance. Ivan et al. [132] introduced a two-stage inverse modeling approach: genetic algorithms adjusted Reduce-Strain-Closure model coefficients, and artificial-neural-network surrogates accelerated calibration against X-ray CT and tensile data. This data-driven workflow halved orientation-prediction errors and improved elastic-modulus and tensile-strength forecasts by 43% and 59%, respectively.

Surrogate modeling of defects and performance has also gained traction. Mendikute et al. [133] generated synthetic low-velocity impact datasets via validated FE models and trained random-forest regressors to predict force–, displacement–, and energy–time curves with $R^{2}>0.995$ in under 5 s per sample, enabling online structural health monitoring. Causon et al. [134] extended this concept to Bayesian inversion in RTM: by partitioning the flow domain and training a neural surrogate, they embedded the surrogate within an ensemble Kalman inversion loop to estimate local permeability and porosity in real time (≤1 s), complete with confidence intervals on predicted defect risks.

Together, these studies exemplify a shift toward hybrid physics--ML frameworks—combining high-fidelity simulations, multi-modal sensing, and advanced learning algorithms—to achieve rapid, reliable predictions and adaptive control in composite manufacturing. Table 2 summarizes recent advances in ML applications across the design, manufacturing, and characterization of fiber-reinforced composites. These works illustrate a broad range of ML integration strategies—including surrogate modeling, image-based recognition, sensor fusion, and hybrid physics–ML frameworks—spanning diverse domains such as material selection, defect detection, process optimization, and real-time monitoring. Key highlights include applications of deep learning for in-line inspection, Bayesian optimization for material design, and surrogate-assisted simulations for high-speed process emulation.

## 4. In-Depth Research Analysis

To enable a structured comparison, the reviewed studies are summarized in Table 3. The feasibility assessments are justified based on three factors: (i) maturity of the ML methodology and dataset size, (ii) extent of experimental or industrial validation, and (iii) integration potential within existing composite manufacturing workflows. Approaches such as digitalized pultrusion frameworks show higher feasibility due to direct compatibility with industrial infrastructure, whereas methods relying on limited datasets or highly specialized equipment remain at medium readiness.

### 4.1. XPBD-Based Surrogate for RL-Driven Robotic Draping

Blies et al. [61] reduced the computational cost of finite element (FE) draping by developing an Extended Position-Based Dynamics (XPBD) surrogate, enabling reinforcement learning (RL) for robotic draping. In FE, stable time steps scale as

$$\Delta t_{\mathrm{stable}}=\frac{L_{e}}{\sqrt{E/\rho }},$$

which becomes prohibitively small for fine meshes (e.g., 8192 triangles on a 370×370 mm blank). XPBD reformulates fabric mechanics as constraints (stretch, bending, shear, contact) solved by positional corrections, permitting larger Δt.

Material parameters were characterized experimentally: tensile tests yielded in-plane modulus $E_{\|}$ and strength $\sigma_{u}$; ±45° shear tests gave shear modulus *G*; bending stiffness $D=Et^{3}/\left[12\left(1-\nu^{2}\right)\right]$ came from three-point bending; and friction coefficients $\mu_{s},\mu_{k}$ were measured with a sled. These values parameterized XPBD constraints.

The surrogate represented the blank as nodes $\left\{p_{i}\right\}$ with mass $m_{i}$, subject to stretch

$$C_{s,ij}=∥p_{i}-p_{j}∥-L_{0,ij},$$

bending $C_{b}=\arccos\left(n_{a}\cdot n_{b}\right)-\theta_{0}$, and contact $C_{c,i}=\phi \left(p_{i}\right)\geq 0$. Constraint stabilization with compliance α allowed larger time steps. Validation against 11 diaphragm-forming experiments confirmed fidelity in fiber orientation θ(x) and displacement fields.

For RL, the state was a 65×65×3 tensor encoding drape status, distance-to-tool, and regrasp indicators. Actions selected target nodes for local adjustment (4225 choices). The sparse reward was

$$R=-∥\Delta_{\mathrm{shape}}{∥}_{2}-w_{o}∥\Delta \theta {∥}_{2}-w_{c}C_{\mathrm{wrinkle}}.$$

PPO training on the XPBD surrogate enabled fast policy learning, later transferred to a real robot with residual correction.

**Figure 7:**
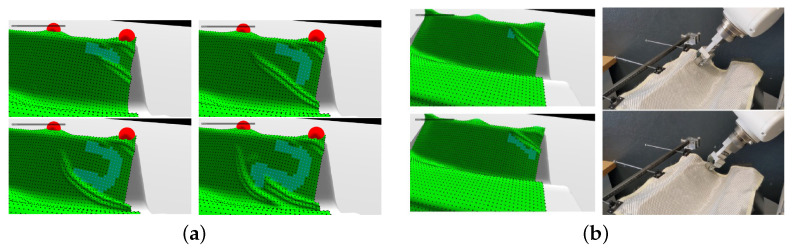
(**a**) Visualization of a manually draped trajectory in our simulation environment; (**b**) snapshots of the optimized trajectory that was obtained by the RL agent and its playback on the real-world robot [61].

The trained policy was transferred to a real collaborative robot (Figure 7b): positional commands matched the surrogate’s coordinate frame, and sensory feedback (vision or force) verified that real drape matched the surrogate-predicted behavior. Discrepancies $\epsilon =∥p_{\mathrm{real}}-p_{\mathrm{sim}}∥$ triggered minor online corrections via a residual policy trained with domain randomization. The success demonstrated that the XPBD surrogate, with its constraint-based formulation and validated material parameters (e.g., $E_{\|},G,D,\mu$), provided sufficient fidelity and speed to train RL policies for complex draping tasks, automating what was traditionally manual and costly (Figure 7a).

This approach overcomes FE bottlenecks, providing validated orientation and wrinkle predictions at interactive speeds. Remaining challenges include accurate mapping of continuum properties to discrete constraints, shear/wrinkling fidelity, and sim-to-real transfer. Extensions to anisotropic multilayer fabrics and advanced representations (e.g., graph networks) are promising directions [137].

### 4.2. HSI-Guided Prediction of Laser-Structured FRP for Metallic Coating Adhesion

Gebauer et al. [138] studied how laser structuring improves adhesion of metallic coatings on FRPs, combining process modeling, hyperspectral imaging (HSI), and deep learning. The pulsed laser fluence

$$F=\frac{E_{p}}{A_{\mathrm{spot}}},\;d_{\mathrm{ab}}=\delta \ln\;\left(\frac{F}{F_{\mathrm{th}}}\right)$$

governed ablation depth, with three regimes: insufficient ($F<F_{\mathrm{th}}$), optimal ($F\approx F_{\mathrm{th}}$), and damaging ($F\gg F_{\mathrm{th}}$). Optimal structuring generated micro-roughness ($R_{a}$) that enhanced adhesion strength $\sigma_{\mathrm{adh}}\propto \tau_{0}+kR_{a}^{\alpha }$. Thermal modeling ensured peak temperature remained below resin decomposition ($T_{\mathrm{decomp}}∼400–500$ °C), preventing fiber damage.

**Figure 8:**
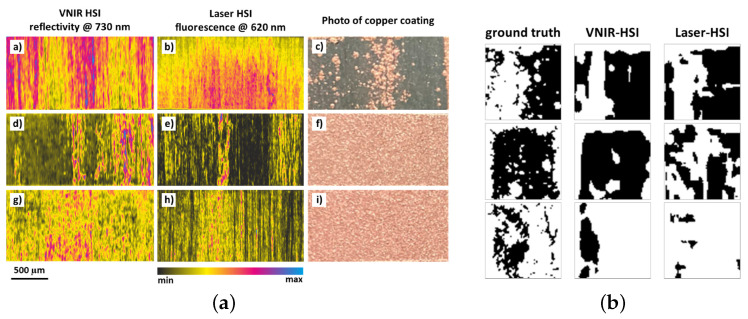
(**a**) HSI and copper coating adhesion for three laser regimes (insufficient, optimal, damaging); (**b**) ground truth vs. U-Net predictions of adhesion quality [138].

HSI captured spectral cubes R(x,y,λ) of structured surfaces (Figure 8A). A modified U-Net segmented regions into {insufficient, optimal, damaging}, using spectral augmentation to improve robustness. Predictions correlated with pull-off adhesion tests of copper coatings ($\sigma_{\mathrm{coat}}=F_{\mathrm{pull}}/A$), achieving ∼80% accuracy (Figure 8B).

The integration of laser processing, HSI classification, and selective rework formed a closed-loop workflow. After an initial laser pass, HSI predicted local adhesion quality; suboptimal regions were selectively re-processed by adjusting fluence or scan patterns. This reduced waste

$$W_{r}=1-\frac{N_{\mathrm{rework}}}{N_{\mathrm{reject}}+N_{\mathrm{rework}}},$$

while ensuring coating quality.

This closed-loop strategy demonstrated how combining ablation physics, HSI, and ML segmentation enables predictive manufacturing of metal-coated FRPs with improved adhesion and reduced scrap.

### 4.3. CNN-Driven Surrogate Optimization for Variable-Geometry Draping

Zimmerling et al. [139] integrated convolutional neural networks (CNNs) into surrogate-based optimization (SBO) for textile draping across varying geometries. A finite-element model (FEM) S(p,G) mapping process parameters p and geometry *G* to quality *Q* is computationally costly. They trained a CNN surrogate ${\hat{S}}_{\theta }\left(p,G\right)$ via reinforcement learning (RL) to predict near-optimal p for new geometries.

Geometries *G* were represented as images $I_{G}\in R^{H\times W\times C}$ encoding tool boundaries, distances, and curvature. The encoder $E_{\phi }\left(I_{G}\right)$ produced latent embeddings *z*, fed to actor $\pi_{\psi }\left(z\right)$ to output pad positions p. Surrogate rewards approximated FEM costs using low-fidelity models:

$$R=-[\alpha E_{w}\left(p,G\right)+\beta D\left(p,G\right)],$$

with $E_{w}$ approximating wrinkle energy and *D* being displacement error. An error predictor $f_{\chi }\left(z,p\right)$ corrected surrogate deviations, limiting FEM calls via trust-region checks. PPO trained actor–critic networks efficiently across geometry distributions G.

**Figure 9:**
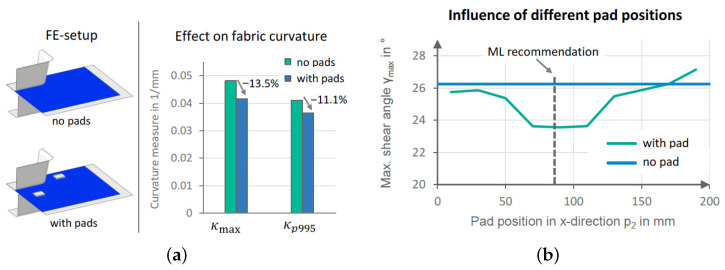
(**a**) Simulation setup and surrogate validation; (**b**) effect of pad positions on maximum shear angle during forming [139].

Validation showed <5% relative error $\epsilon =|S\left(p^{*},G\right)-\min_{p}S\left(p,G\right)|/\min_{p}S\left(p,G\right)$ on novel box-shaped geometries. Sensitivity analysis via $\nabla_{z}\pi_{\psi^{*}}\left(z\right)$ identified regions needing FEM refinement (Figure 9b). Advantages include rapid prediction of near-optimal pad placements and reduced computational cost, while challenges involve surrogate accuracy and generalization to complex 3D surfaces.

Future work should extend the method to non-parametric 3D surfaces, improve wrinkle surrogate physics, and incorporate uncertainty-aware rewards and real-time feedback to enhance generalization and robustness.

### 4.4. Digitized Data Acquisition and Synchronization for Pultrusion

**Figure 10:**
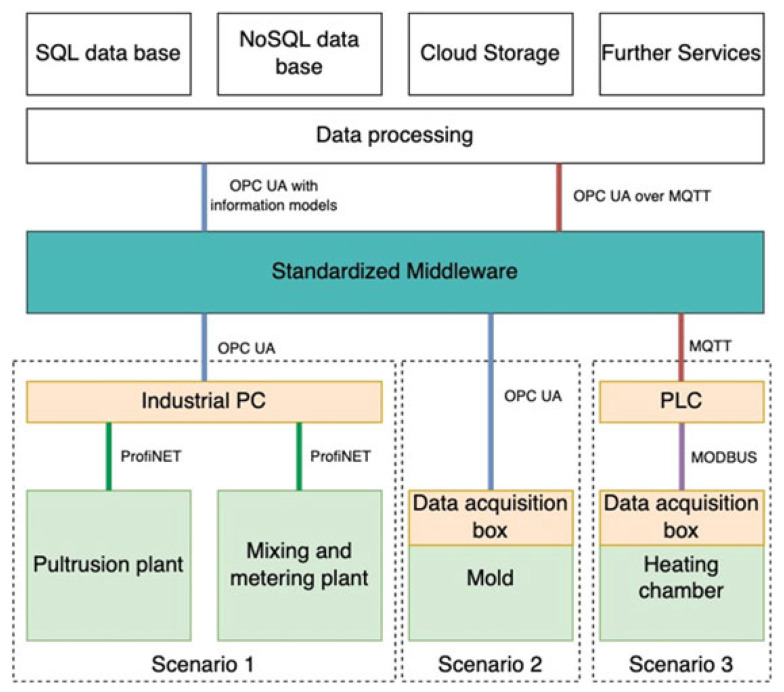
Schematic overview of data acquisition, synchronization, and ML analytics in a pultrusion line [139].

Helfrich et al. [140] developed a standardized framework for real-time data acquisition in pultrusion, enabling closed-loop control of temperature, pull speed, resin viscosity, and structural parameters (Figure 10). Each sensor $s_{i}\left(t\right)$ was sampled at Nyquist-compliant frequency $f_{s,i}\geq 2f_{\max,i}$, with timestamps corrected for network delays $\delta_{i}=t_{\mathrm{recv}}-t_{\mathrm{send}}-\tau /2$ to maintain synchronization $|t_{i}-t_{j}|<\epsilon_{\mathrm{sync}}$. Data throughput $B=\sum_{i}b_{i}f_{s,i}$ was managed via buffers $N_{\mathrm{buf}}\geq B_{\max}T_{\max-\mathrm{lat}}/b_{\mathrm{frame}}$ and low-latency OPC UA/MQTT communication:

$$L=t_{\mathrm{acq}}+t_{\mathrm{proc}}+t_{\mathrm{trans}}+t_{\mathrm{store}}.$$

Dynamic signals were filtered in real time using moving averages or Kalman filters:

$$\hat{x}\left[n|n\right]=\hat{x}\left[n|n-1\right]+K\left[n\right]\left(z\left[n\right]-H\hat{x}\left[n|n-1\right]\right),$$

improving reliability under Gaussian noise. Derived features included thermal gradients $\nabla T\approx (T_{i+1}-T_{i})/\Delta x$, resin viscosity $\eta \left(T\right)=\eta_{0}\exp\left(E_{a}/\left(RT\right)\right)$, and energy input $P_{\mathrm{in}}=F\left(t\right)\cdot v_{p}\left(t\right)$. Slow signals were upsampled or interpolated to align with faster data streams, producing synchronized multivariate time series for ML-based analysis.

The architecture supported secure, low-latency analytics via TLS, hierarchical MQTT topics, and OPC UA nodes. Moving-window anomaly detection employed z-scores or PCA residuals $∥X-\sum_{j}\lambda_{j}v_{j}v_{j}^{⊤}∥$, and LSTM models could predict future trends for predictive maintenance. This standardized, synchronized dataset enables training of surrogate ML models $y=f_{\mathrm{ML}}\left(x\right)+\epsilon$ for real-time optimization and digital twin implementation.

This framework addressed key challenges: precise timestamping $\delta_{i}$, high-throughput buffering, and noise reduction via Kalman filtering, enabling reliable multivariate time series for ML. Future work includes advanced predictive maintenance using LSTMs, real-time control via adaptive Kalman tuning of process noise *Q*, and digital twin integration for optimized pultrusion performance.

### 4.5. Predicting GFRP Fracture Toughness from Standard Mechanical Properties via ML

Karamov et al. [124] developed an ML framework to predict fracture toughness $K_{IC}$ of pultruded GFRP from standard mechanical tests (tensile, flexural, compressive, shear, impact). A total of 600 specimens from 50 batches were tested, with $K_{IC}$ measured via ASTM E399 compact tension (CT) specimens:

$$K_{IC}=\frac{P_{\max}}{B\sqrt{W}}\;f\left(a/W\right),\;G_{IC}=\frac{K_{IC}^{2}}{E^{\prime }},\;E^{\prime }=\frac{E}{1-\nu^{2}}.$$

Input features x included $\sigma_{0{}^{\circ}},\sigma_{90{}^{\circ}},\sigma_{f},\sigma_{c},\tau ,E_{0{}^{\circ}},E_{90{}^{\circ}},E_{f},U_{\mathrm{imp}},V_{f},\rho$, normalized as ${\tilde{x}}_{i}=\left(x_{i}-\mu_{i}\right)/\sigma_{i}$. Pearson correlation analysis and PCA reduced multicollinearity ($|r_{ij}|>0.9$).

Three regressors were trained:

- ANN: input $\tilde{x}$ → hidden layers → linear output $\hat{y}$, optimized with MSE and early stopping.
  $$h^{\left(\ell +1\right)}=\sigma \left(W^{\left(\ell \right)}h^{\left(\ell \right)}+b^{\left(\ell \right)}\right)$$
- Random Forest: ensemble of regression trees, prediction via averaging:
  $$\hat{y}=\frac{1}{T}\sum_{t=1}^{T}T_{t}\left(\tilde{x}\right)$$
- Gradient Boosting (GBDT): additive model handling nonlinear feature interactions:
  $$F_{m}\left(\tilde{x}\right)=F_{m-1}\left(\tilde{x}\right)+\nu \;h_{m}\left(\tilde{x}\right)$$

GBDT achieved the lowest MSE (<0.1${\bar{K_{IC}}}^{2}$), <10% relative error. Partial dependence plots revealed how longitudinal flexural strength $\sigma_{f}$ strongly influences $K_{IC}$, consistent with micromechanics:

$$G_{IC}\approx G_{m}+V_{f}G_{f}+\alpha_{\mathrm{int}}\tau_{\mathrm{int}}\ell$$

**Figure 11:**
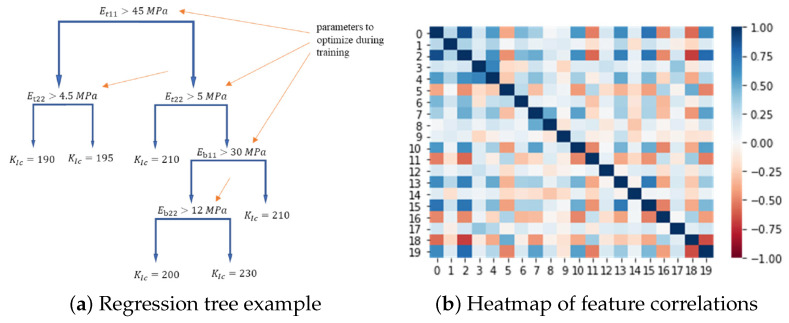
ML-based prediction of GFRP fracture toughness [124].

The model (shown in Figure 11) enables rapid prediction of $K_{IC}$ and derived $G_{IC}$, facilitating virtual material qualification for finite element simulations and reducing reliance on laborious CT tests.

Challenges of Karamov et al.’s ML approach for predicting the fracture toughness ($K_{IC}$) of pultruded GFRP composites from standard mechanical tests include the inherent variability in measured $K_{IC}$ due to microstructural factors like fiber volume fraction ($V_{f}$), interfacial adhesion quality, and process-induced residual stresses, which complicate model generalization. Feature selection required careful handling of multicollinearity (using PCA for features with Pearson correlation $|r_{ij}|>0.9$) to avoid overfitting, and ensuring physical consistency between predictions and micromechanical failure mechanisms (e.g., matrix cracking, fiber pull-out) demanded residual analysis and feature engineering. Advantages are substantial: the methodology bypasses the laborious and expensive ASTM E399 compact tension (CT) test, predicting $K_{IC}$ in milliseconds using readily available inputs like tensile ($\sigma_{0{}^{\circ}},\sigma_{90{}^{\circ}}$), flexural ($\sigma_{f}$), and impact ($U_{\mathrm{imp}}$) properties. The optimized GBDT model achieved high accuracy (<10% relative error, $\mathrm{MSE}<{\left(0.1\bar{K_{IC}}\right)}^{2}$), within experimental scatter, by effectively capturing nonlinear feature interactions through its additive model structure ($F_{m}=F_{m-1}+\nu h_{m}$). Strong correlations identified by the model (e.g., between $\sigma_{f}$ and $K_{IC}$) align with micromechanics, as flexural strength reflects matrix toughness and interfacial shear strength ($\tau_{\mathrm{int}}$), key contributors to $G_{IC}\approx G_{m}+V_{f}G_{f}+\alpha_{\mathrm{int}}\tau_{\mathrm{int}}\ell$. Future directions may include uncertainty quantification via quantile regression forests to provide prediction intervals for engineering design, extending the model to account for environmental degradation (moisture, temperature) or fatigue loading, and incorporating microstructural descriptors (e.g., from imaging) alongside macroscopic properties. Furthermore, integrating the predicted $G_{IC}=K_{IC}^{2}/E^{\prime }$ directly into cohesive zone models for finite element simulations could streamline virtual material qualification, accelerating the development of next-generation composites.

### 4.6. ML-Driven Prediction of Axial Load Capacity in Pultruded GFRP Columns

Kajendran et al. [141] studied axial compression of hollow rectangular pultruded GFRP columns, analyzing how width-to-thickness ratio B/t, aspect ratio H/B, and column height *H* affect ultimate load $P_{u}$ and initial stiffness $K_{\mathrm{init}}$. The cross-sectional area and moment of inertia about the minor axis are approximated as

$$A\approx 2\left(B+H_{\mathrm{cs}}-2t\right)\;t,\;I\approx \frac{BH_{\mathrm{cs}}^{3}-\left(B-2t\right){\left(H_{\mathrm{cs}}-2t\right)}^{3}}{12},$$

with effective longitudinal modulus

$$E_{L}=V_{f}E_{f}+\left(1-V_{f}\right)E_{m}.$$

The slenderness ratio is $\lambda =L_{\mathrm{eff}}/\sqrt{I/A}$, with Euler-type buckling load $P_{\mathrm{cr}}=\pi^{2}E_{L}I/L_{\mathrm{eff}}^{2}$, crushing capacity $P_{\mathrm{crush}}\approx \sigma_{c}A$, and initial stiffness $K_{\mathrm{init}}\approx E_{L}A/H$. Observed failure modes ranged from uniform crushing at low λ to global buckling or longitudinal splitting at corners due to transverse tensile stresses $\sigma_{⊥}$ approaching the matrix tensile strength $\sigma_{m,t}$.

To predict $P_{u}$ from geometric ratios, Kajendran et al. applied a second-order RSM polynomial in B/t, H/B, and *H* achieving $R^{2}=0.8347$, capturing general trends but not nonlinear interactions. An ANN model normalized the inputs ${\tilde{x}}_{i}=\left(x_{i}-\mu_{i}\right)/\sigma_{i}$ and propagated them through hidden layers

$$h^{\left(\ell +1\right)}=\sigma \left(W^{\left(\ell \right)}h^{\left(\ell \right)}+b^{\left(\ell \right)}\right),\;h^{\left(0\right)}={\left[{\tilde{x}}_{1},{\tilde{x}}_{2},{\tilde{x}}_{3}\right]}^{⊤},$$

to output $\hat{P_{u}}$. The ANN achieved correlation R>0.688 across training and validation, outperforming RSM. Partial derivatives $\partial \hat{P_{u}}/\partial \left(B/t\right)$ and $\partial \hat{P_{u}}/\partial \left(H/B\right)$ confirmed that increasing either ratio reduces ultimate load, consistent with experimental observations. The ANN implicitly captured transitions between crushing-dominated failure ($P_{u}\approx \sigma_{c}A$) and slenderness-controlled instability ($P_{u}\approx P_{\mathrm{cr}}$).

This ML surrogate enables rapid estimation of $P_{u}$ and $K_{\mathrm{init}}$, guiding cross-sectional design via

$$\hat{P_{u}}\left(B/t,H/B,H\right)\geq P_{\mathrm{req}},$$

and informing FEM parameters for larger structural analyses. Challenges include capturing the nonlinear interplay of slenderness, local wall stability, and corner stress concentrations with limited experimental data, while advantages include efficient mapping of simple geometric ratios to load capacity, reducing reliance on costly tests. Future work should expand the dataset, incorporate stress concentration factors to better predict splitting, model post-peak damage, and integrate the surrogate with topology optimization for lightweight pultruded sections.

### 4.7. Performance Comparison of ML Models

CNNs and their 3D/spectral variants dominate imaging and hyperspectral tasks because their convolutional inductive bias captures local spatial–spectral structure efficiently. In HSI-based adhesion classification and surface-defect segmentation CNN/U-Net models produced practical accuracies (80%) and enabled spatially resolved rework maps [138], while geometry-to-policy CNN encoders in surrogate–RL frameworks produced near-optimal pad placements with relative errors <5% on held-out shapes [139]. These high numbers occur because CNNs exploit strong locality and translation equivariance in images and can learn rich hierarchical features when many labeled examples or realistic augmentations exist; however, they demand substantial labeled data, GPU computing, and careful augmentation or pretraining to avoid overfitting in industrial settings [110,114].

When spectral or volumetric channels are included (HSI, XCT), the input dimensionality rises sharply and so do model size, memory footprint, and inference latency; naively training 3D or spectral-aware networks therefore becomes computationally expensive and vulnerable to overfitting unless constrained by domain priors. In practice, it is recommendable to follow several complementary measures: enforce physics-informed regularizers (spectral-smoothness, BRDF-aware losses, and temperature/energy conservation terms) to bias learning toward physically plausible solutions [142]; reduce runtime and memory by patch/tiling strategies [143], mixed-precision inference [144], pruning or knowledge-distillation [145] to compact models, and by using lightweight encoders [146] (e.g., 2D spatial backbones with shallow spectral heads [147]) when throughput matters. Preprocessing choices strongly affect robustness—reflectance calibration, continuum removal, Savitzky–Golay smoothing, explicit band-selection or PCA, and spectral augmentation (Gaussian noise, small wavelength shifts, band dropout) must be reported because they change signal-to-noise and can create modality-specific artifacts that the network will exploit.

Gradient boosting decision trees produced the best tabular predictions in materials property tasks; for example, GBDT gave under 10 percent relative error for fracture toughness on a 600 specimen dataset. [124] Tree ensembles succeed here because they capture nonlinear feature interactions with little preprocessing and are robust to heterogeneous feature scales and moderate sample sizes [124].

Random forests matched GBDT within a few percentage points on noisy tabular data and served as a resilient baseline with fast uncertainty estimates. [114,124] This robustness is due to bootstrap aggregation that reduces variance and produces interpretable feature importance metrics without heavy hyperparameter tuning. [114]

Error propagation in industrial ML pipelines depends strongly on model type, data modality, and available training resources. CNNs, while highly accurate on imaging and hyperspectral tasks, propagate errors through high-dimensional hierarchical features, making them sensitive to domain shifts and input noise [148], which can amplify prediction deviations if not mitigated by augmentation or physics-informed regularization. Tree ensembles, such as GBDT and random forests, localize errors through split-based decisions and ensemble averaging [149], producing naturally bounded uncertainty even with smaller datasets. Hybrid physics-informed models combine these strengths, constraining outputs to physically plausible ranges and reducing error accumulation [150], making them particularly effective in small-data or extrapolative industrial scenarios.

Interpretability and uncertainty quantification follow complementary patterns. Tree ensembles (GBDT, RF) produce natural, actionable interpretability (feature ranking, PDPs) and support built-in uncertainty proxies (quantile forests, bootstrap intervals), making them preferable when traceability and conservative design bounds are required [106,124]. CNNs require post hoc explainability (Grad-CAM, saliency, SHAP on embedded features) and calibrated uncertainty (MC dropout, ensembles) to reach comparable trust for safety-critical process control; several works combined SHAP/GAN augmentation to probe feature importance in small-sample regimes [4,110].

Data requirements and robustness differ sharply. For imaging tasks, CNNs typically need thousands of labeled frames or reliable synthetic/simulated augmentation (e.g., FE-derived images, GAN augmentation) to generalize; when such augmentation was used, models achieved industrially useful speed/accuracy trade-offs (e.g., ultrasonic PAUT pipelines and synthetic leak data) [107,114]. For process/property prediction from engineered features, GBDT/RF reached strong performance with hundreds of well-curated samples (Karamov’s 600 specimens; Perin’s 1000 simulations), explaining why practitioners prefer tree methods for small to medium tabular datasets [105,124,151].

Computational cost and runtime constraints also guide method choice. CNNs and deep surrogates (deep-material networks, PixelRNN) deliver real-time inference for images when deployed on GPUs and are suitable for inline vision systems, but training and hyperparameter tuning are expensive [111,136]. Tree ensembles train quickly on CPUs and are easy to deploy in embedded controllers for rapid decision rules or surrogate cost prediction; they are therefore attractive for digital-twin or edge scenarios with limited hardware [105,124].

Hybrid and physics-informed solutions reduce data hunger and improve extrapolation: physics-augmented GNN/XPBD surrogates and multi-fidelity Bayesian loops embedded physical constraints that lowered required labels for control and design tasks [61,104,111]. Where available, these hybrids achieved both accuracy and interpretability because the physics priors constrained feasible outputs and reduced the effective hypothesis space, explaining why many recent high-impact studies paired ML with simulation or analytic rules [104,111].

In practice, model selection should be guided by data modality and operational needs: CNNs (or vision transformers) for high-resolution imaging/HSI tasks when annotated image volumes (or validated synthetic generators) exist [110,138]; GBDT/RF for tabular process/property prediction, rapid surrogate building, and interpretable root-cause analysis [105,124]; and hybrid physics–ML surrogates when extrapolation, control stability, or small-data generalization are central (XPBD-GNN, multi-fidelity BO) [61,104]. The observed performance gaps therefore follow from (i) the match between model inductive bias and data structure (local filters vs. feature splits), (ii) available sample size and augmentation/transfer mechanisms, and (iii) the need for uncertainty/interpretability in industrial deployment.

## 5. AI Model Architecture for Natural Fiber-Based Composite Fabrication 

Building upon the methodologies reviewed in XPBD-based RL draping [61] and HSI-based surface prediction [138], a hybrid AI model is proposed tailored to natural fiber-reinforced polymer (NFRP) [152] composite fabrication. The model integrates physics-informed simulation with vision-based inspection to guide process automation and quality control.

The implementation of this hybrid AI stack aims to translate laboratory concepts into industrial practice. Its goal is to enable automated forming, real-time defect detection, process optimization, and reduced scrap in natural fiber-reinforced composite production. At the core is a digital twin of the manufacturing line, where physics-informed surrogates provide reliable initial predictions, and trainable modules refine them using real-time sensor and hyperspectral data.

Transitioning to pilot or production deployment requires standardized data pipelines, including temporal synchronization and spectral calibration, multi-fidelity training and domain translation, uncertainty quantification, and safe action-limiting mechanisms. Expected benefits include faster process setup, shortened experimental cycles, and lower material waste. Key engineering challenges involve limited training datasets, sim-to-real discrepancies, computational demands, and interdisciplinary coordination. Deployment is best approached incrementally, moving from laboratory pilots to controlled production lines and finally to full-scale operations, with continuous model validation and built-in fallback procedures to ensure reliability and robustness.

### 5.1. Hybrid XPBD–GNN Surrogate for Forming Simulation

To capture the anisotropic, nonlinear, and moisture-sensitive response of natural fibers, the XPBD mass–spring surrogate is augmented with a physics-informed GNN. The preform is discretized into a graph G=(V,E), where each node i∈V represents a patch of fibers characterized by

$$h_{i}=\left[E_{\|,i},\;G_{i},\;D_{i},\;\mu_{s,i},\;\mu_{k,i},\;\phi_{i},\;\alpha_{i},\;t_{i}\right],$$

with

$$E_{\|,i}=\frac{d\sigma_{i}}{d\varepsilon }{|}_{\varepsilon \to 0},\;G_{i}=\frac{\tau_{i}}{\gamma_{i}},\;D_{i}=\frac{E_{\|,i}\;t_{i}^{3}}{12(1-\nu^{2})},$$

and moisture adsorption modeled by

$$\phi_{i}:\;\frac{\partial \phi }{\partial t}=D_{\phi }\;\nabla^{2}\phi ,\;\alpha_{i}=\alpha_{0}\;\phi_{i},$$

where $D_{\phi }$ is the diffusion coefficient and $\alpha_{i}$ the local swelling coefficient. Edges encode adjacency and orientation mismatch:

$$e_{ij}=\left[∥p_{i}-p_{j}∥,\;\cos\left(\theta_{i}^{0}-\theta_{j}^{0}\right)\right].$$

Within each XPBD time step Δt, tentative positions $p_{i}^{\prime }$ are first computed by

$$p_{i}^{\prime }=p_{i}+\Delta t\;v_{i}+\Delta t^{2}\;g,$$

then corrected via constraint projection (e.g., stretch $C_{s,ij}$, bending $C_{b}$, contact $C_{c,i}$) to yield $p_{i}^{n+1}$ per

$$\Delta p_{i}=-\sum_{c}\frac{\lambda_{c}}{m_{i}}\nabla_{p_{i}}C_{c},\;\lambda_{c}=-\frac{C_{c}\left(p^{\prime }\right)}{∥\nabla C_{c}{∥}^{2}+\alpha_{c}/\Delta t^{2}}.$$

Concurrently, the GNN refines nodal states via *L* layers of message passing:

$$h_{i}^{\left(l+1\right)}=\sigma \;\left(W_{0}\;h_{i}^{\left(l\right)}+\sum_{j\in N(i)}W_{1}\;e_{ij}+W_{2}\;h_{j}^{\left(l\right)}\right),$$

embedding physics through a loss term

$$L_{\mathrm{phys}}=\sum_{i}{∥\;\nabla \;\cdot {\left(k_{i}\nabla T\right)}_{i}∥}^{2}+\sum_{\left(i,j\right)}{∥\;{\hat{L}}_{0,ij}-L_{ij}^{\mathrm{ref}}∥}^{2},$$

where $k_{i}=k\left(\phi_{i},E_{\|,i}\right)$ is effective thermal conductivity, and $L_{ij}^{\mathrm{ref}}$ is the rest length from high-fidelity data. Finally, the surrogate outputs local deformation gradients $F_{i}$ and fiber reorientation $\Delta \theta_{i}=\arccos\left(\frac{F_{i}e_{i}^{0}\cdot t_{ij}}{∥F_{i}e_{i}^{0}∥∥t_{ij}∥}\right)$, which feed into the RL-based forming controller.

### 5.2. HSI-Enhanced Surface Defect Segmentation

For post-process evaluation of NFRP surfaces (e.g., fiber distribution, surface fuzz, resin pooling), a physics-guided spectral-UNet [153] is employed that processes calibrated reflectance cubes

$$I\left(x,y,\lambda \right)=\frac{I_{\mathrm{raw}}\left(x,y,\lambda \right)-I_{\mathrm{dark}}\left(\lambda \right)}{I_{\mathrm{white}}\left(\lambda \right)-I_{\mathrm{dark}}\left(\lambda \right)}\;,$$

yielding $I\in R^{H\times W\times B}$. Prior to network input are applied 1. continuum removal to normalize cellulose and lignin absorption features:

$$R_{\mathrm{cr}}\left(\lambda \right)=\frac{R\left(\lambda \right)-R_{\mathrm{convex}}\left(\lambda \right)}{R_{\mathrm{convex}}\left(\lambda \right)}\;,$$

2. Savitzky–Golay smoothing of each spectrum s(λ) to reduce sensor noise,

3. spectral derivative $\frac{dR}{d\lambda }$ to enhance narrow absorption peaks (e.g., O–H stretch at 1.4 µm).

The Spectral-UNet uses 3D convolutions across spatial and spectral axes:

$$F_{\mathrm{enc}}^{\left(l+1\right)}=\sigma \left(W^{\left(l\right)}{*}_{3D}F_{\mathrm{enc}}^{\left(l\right)}+b^{\left(l\right)}\right),\;F_{\mathrm{dec}}^{\left(l\right)}=\mathrm{UpConv}\left(F_{\mathrm{enc}}^{\left(l+1\right)}\right)⊕F_{\mathrm{enc}}^{\left(l\right)},$$

where ⊕ denotes channel-wise concatenation and σ a nonlinear activation (e.g., LeakyReLU). To enforce both spatial and spectral coherence, the following physics-informed regularizers are included:

$$L_{\mathrm{spec}-\mathrm{smooth}}=\sum_{x,y}\sum_{b=2}^{B-1}{\left|R\left(x,y,\lambda_{b+1}\right)-2R\left(x,y,\lambda_{b}\right)+R\left(x,y,\lambda_{b-1}\right)\right|}^{2},$$

$$L_{\mathrm{spat}-\mathrm{smooth}}=\sum_{b=1}^{B}\sum_{x,y}{∥\nabla_{xy}\;p_{c}\left(x,y;\lambda_{b}\right)∥}^{2},$$

where $p_{c}\left(x,y;\lambda_{b}\right)$ are per-band class probabilities.

The final segmentation into

c∈{voids,richregions,fuzz,wrinkles,good}

is obtained by a softmax along the class dimension, producing $p_{c}\left(x,y\right)$. Training minimizes the combined loss:

$$L=L_{\mathrm{CE}}+\lambda_{\mathrm{Dice}}\;L_{\mathrm{Dice}}+\lambda_{\mathrm{KL}}\;D_{\mathrm{KL}}\left(p_{c}∥q_{c}\right)+\lambda_{\mathrm{spec}}L_{\text{spec-smooth}}+\lambda_{\mathrm{spat}}L_{\text{spat-smooth}},$$

where $q_{c}$ is a soft ground-truth distribution capturing class uncertainty in naturally variable fiber textures.

### 5.3. Multi-Modal Fusion for Closed-Loop Control

Real-time, closed-loop optimization of NFRP processing is achieved by fusing the global forming state from the XPBD–GNN surrogate with surface quality metrics from the Spectral-UNet via a cross-attention mechanism. The global nodal embedding

$$h_{\mathrm{GNN}}=\frac{1}{\left|V\right|}\sum_{i\in V}h_{i}^{\left(L\right)}$$

and the hyperspectral summary vector

$$s_{\mathrm{HSI}}=\frac{1}{HW}\sum_{x,y}{\left[p_{\mathrm{good}}\left(x,y\right),\;p_{\mathrm{void}}\left(x,y\right),\;p_{\mathrm{fuzz}}\left(x,y\right)\right]}^{⊤}$$

are linearly projected to obtain query, key, and value embeddings:

$$q=W_{q}\;h_{\mathrm{GNN}},\;k=W_{k}\;s_{\mathrm{HSI}},\;v=W_{v}\;s_{\mathrm{HSI}}.$$

Attention weights are computed via

$$A=\mathrm{softmax}\;\left(\frac{q^{⊤}k}{\sqrt{d_{k}}}\right),$$

and the fused representation is formed as

$$h_{\mathrm{attn}}=A\;v,\;z=\mathrm{LayerNorm}\left(h_{\mathrm{GNN}}+h_{\mathrm{attn}}\right).$$

A multilayer perceptron then maps z to control adjustments

$$a_{t}={\left[\Delta P_{\mathrm{comp}},\;\Delta T_{\mathrm{zone}},\;\Delta R_{\mathrm{rate}}\right]}^{⊤},$$

which are projected onto allowable bounds

$$a_{t}^{\mathrm{safe}}=\arg\min_{u\in U}{∥u-a_{t}∥}_{2}^{2},\;U=\left[P_{\min},P_{\max}\right]\times \left[T_{\min},T_{\max}\right]\times \left[R_{\min},R_{\max}\right].$$

The instantaneous reward is defined as

$$r_{t}=-\left({\alpha \;∥\Delta p∥}_{2}^{2}+\beta \left(1-\bar{p_{\mathrm{good}}}\right)+\gamma \;\frac{E_{\mathrm{consumed}}}{E_{\max}}\right),$$

with

$${∥\Delta p∥}_{2}={∥p_{\mathrm{sim}}-p_{\mathrm{target}}∥}_{2},\;\bar{p_{\mathrm{good}}}=\frac{1}{HW}\sum_{x,y}p_{\mathrm{good}}\left(x,y\right),$$

and $E_{\mathrm{consumed}}$ estimating energy usage. A temporal smoothness regularizer

$$L_{\mathrm{smooth}}=\rho \;{∥a_{t}^{\mathrm{safe}}-a_{t-1}^{\mathrm{safe}}∥}_{2}^{2}$$

penalizes abrupt transitions. This attention-based fusion ensures that global structural predictions and localized surface assessments jointly inform control decisions, resulting in consistent composite quality under varying process conditions.

### 5.4. Transfer Learning and Domain Randomization

To address natural-fiber variability (e.g., differences in cellulosic microstructure [154], lumen content [155], and moisture uptake between flax and hemp [156]), the following strategies are employed:

- A residual policy $\Delta \pi_{\psi }\left(s\right)$ is learned via proximal policy optimization (PPO) on real-world rollouts, correcting the model-based controller $\pi_{\mathrm{MB}}$:
  $$\pi_{\mathrm{real}}\left(s\right)=\pi_{\mathrm{MB}}\left(s\right)+\Delta \pi_{\psi }\left(s\right),$$
  where the PPO objective includes a sim-to-real discrepancy penalty
  $$L_{\mathrm{PPO}}=E\left[\min\left(r_{t}\left(\psi \right)\;A_{t},\;\mathrm{clip}\left(r_{t}\left(\psi \right),1-\epsilon ,1+\epsilon \right)\;A_{t}\right)\right]+\lambda_{\Delta }\;{∥\Delta \pi_{\psi }\left(s\right)∥}_{2}^{2}.$$
- A CycleGAN [157] is trained to map lab-calibrated reflectance spectra $R_{\mathrm{lab}}\left(\lambda \right)$ to production-domain spectra $R_{\mathrm{prod}}\left(\lambda \right)$, preserving key absorption features (e.g., O–H, C–H bands):
  $$L_{\mathrm{cyc}}=∥G\left(F\left(R_{\mathrm{lab}}\right)\right)-R_{\mathrm{lab}}{∥}_{1}+{∥F\left(G\left(R_{\mathrm{prod}}\right)\right)-R_{\mathrm{prod}}∥}_{1},$$
  $$L_{\mathrm{spec}}=\sum_{\lambda }{\left|R_{\mathrm{lab}}\left(\lambda \right)-R_{\mathrm{prod}}\left(\lambda \right)\right|}^{2},$$
  where G:lab→prod and F:prod→lab. This ensures the Spectral-UNet sees realistic production spectra.
- During surrogate training, material parameters are sampled from distributions reflecting natural-fiber heterogeneity:
  $$E_{\|}∼N\left(\mu_{E},\sigma_{E}^{2}\right),\;G∼N\left(\mu_{G},\sigma_{G}^{2}\right),\;\mu_{s/k}∼\mathrm{Uniform}\left(\left[\mu_{\min},\mu_{\max}\right]\right),$$
  $$\phi_{\mathrm{init}}∼\mathrm{Beta}\left(\alpha_{\phi },\beta_{\phi }\right),\;D_{\phi }∼\mathrm{LogNormal}\left(\mu_{D},\sigma_{D}^{2}\right).$$
  Each XPBD–GNN rollout uses a random draw $\left(E_{\|},G,\mu_{s},\phi ,D_{\phi }\right)$ to enforce robustness. An auxiliary consistency loss penalizes extreme deviations from reference data:
  $$L_{\mathrm{rand}}=\sum_{i}{∥{\hat{y}}_{i}\left(\vartheta \right)-y_{i}^{\mathrm{ref}}∥}^{2},$$
  where ϑ denotes the randomized parameter set.

This integrated model combines physics-based simulation (XPBD–GNN), hyperspectral vision (Spectral-UNet), and RL-based control, specifically tuned for the challenges of NFRP fabrication. The modular architecture enables generalization across fiber types, forming geometries, and quality metrics.

### 5.5. Model Inference

To demonstrate end-to-end functionality, a dummy case was constructed using randomly generated data that mimics key aspects of a natural-fiber composite process:

- Graph input:
  – N=10 nodes sampling local patches of the fiber preform, each with an 8-dimensional feature vector $x_{i}∼N\left(0,1\right)$ (e.g., local modulus, shear, moisture, thickness).
  – E=20 random undirected edges (i,j), with 2-dimensional edge attributes $e_{ij}∼N\left(0,1\right)$ (e.g., relative distance and orientation).
  – 3D positions $p_{i}∼N{\left(0,1\right)}^{3}$ used only for visualization.
- Hyperspectral image (HSI) input:
  – A single hyperspectral cube of shape [B=50,H=32,W=32,D=8], with reflectance values drawn from a standard normal distribution to emulate pre-processed spectral bands.
- Expected outputs:
  1. *XPBD*-GNN surrogate predicts per-node deformation gradients $F_{i}\in R^{2\times 2}$ (flattened to four values) and a scalar reorientation $\theta_{i}$. In the dummy run, we observe
    $$F_{\mathrm{pred}}:\left(10\times 4\right),\;\theta_{\mathrm{pred}}:\left(10\times 1\right).$$
  2. *Spectral*-UNet produces a volumetric segmentation logits tensor of shape [1,5,32,32,8], corresponding to five material/defect classes across the HSI volume.
  3. *Cross*-Attention Controller fuses the graph embedding (averaged hidden state, dim=16) and a summary HSI feature vector (e.g., fractions of “good,” “void,” “fuzz”) to output three control adjustments:
    $$\left[\Delta P_{\mathrm{comp}},\;\Delta T_{\mathrm{zone}},\;\Delta R_{\mathrm{rate}}\right]\approx \left[0.25,\;0.06,\;0.32\right].$$

This synthetic pipeline validates that each module—physics-informed GNN, volumetric segmentation network, and attention-based controller—integrates correctly: random inputs propagate through the XPBD surrogate, HSI-UNet, and controller, producing outputs of the right shape and range. In a real application, one would replace the dummy data with actual preform measurements, calibrated spectral scans, and deploy the learned controller to adjust compaction pressure, temperature profile, and resin feed in real time.

**Figure 12:**
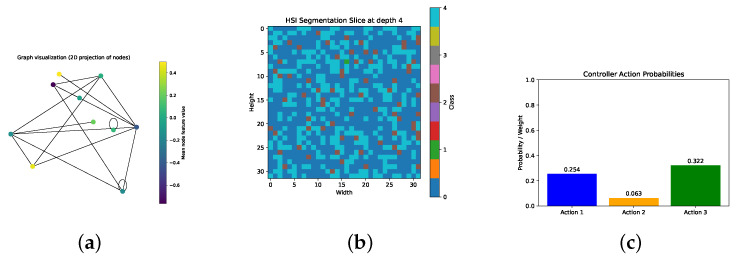
(**a**) Graph topology and node features; (**b**) HSI segmentation slice at depth 4; (**c**) controller action weights.

Figure 12a is a graph illustrating the structural and functional connectivity between different regions of the system. The node color gradient reveals variations in local conditions or material properties, which can point to inhomogeneities or stress zones. Such visualization is instrumental for localized diagnosis, targeted interventions, and adaptive control decisions. A graph-based representation of the composite domain is where nodes correspond to sampled regions or monitored points and edges encode physical or topological relations (e.g., spatial proximity or thermal conductance). The color of each node reflects the mean value of its input features (e.g., local temperature, moisture, or pressure indicators).

Figure 12b shows the result of HSI segmentation at depth z=4 within the 3D volume. Each pixel is assigned to one of the material classes predicted by the Spectral-UNet model. The colormap (tab10) visualizes the predicted categories, which may correspond to: 0: void, 1: rich resin, 2: fuzz, 3: wrinkle, 4: good material. The HSI segmentation provides spatially resolved classification across the volume, which is essential for assessing resin distribution quality and detecting potential manufacturing defects (e.g., wrinkles or voids) prior to curing. The depth slice allows localization of issues at specific composite thickness layers, enhancing process understanding and control.

Figure 12c is the bar chart that depicts the output values of the CrossAttentionController, which fuses features from the GNN and HSI input to guide process adjustments. Each bar corresponds to one of the three process control parameters: δP—compaction pressure adjustment, δT—temperature change, δR—resin addition/removal. The controller output provides interpretable suggestions for adjusting process parameters. In this case, the dominant value is for ΔR, indicating a potential need to alter resin content in the affected region. The controller executes an adaptive strategy based on both internal latent states and real-time visual/material information, enabling proactive correction.

## 6. Discussion

**Figure 13:**
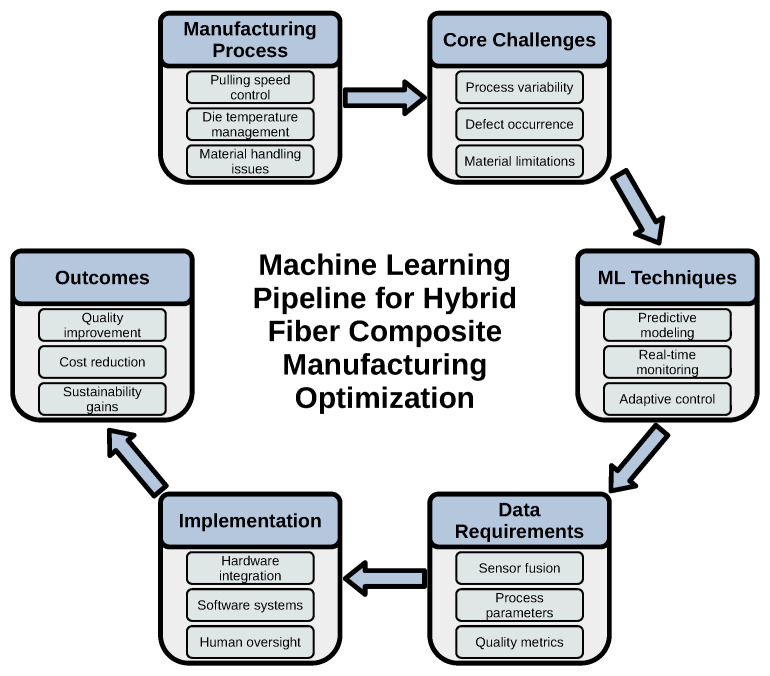
High-level schematic of ML pipeline for hybrid fiber composite manufacturing.

The studies surveyed demonstrate several notable strengths. First, by combining rich experimental or simulation data with modern ML architectures, they often achieve predictive accuracies and execution speeds unattainable by purely physics-based or rule-based methods. Figure 13 presents a high-level overview of the ML pipeline designed to address key challenges in hybrid fiber composite manufacturing. The pipeline spans the full process—from core physical and material challenges to actionable outcomes—illustrating how ML techniques can be integrated with domain-specific knowledge and real-time data acquisition. Each stage includes critical inputs such as process parameters, sensor data, and human oversight and leads to targeted outcomes such as improved quality, reduced costs, and enhanced sustainability. This systems-level perspective forms the foundation for building interpretable, adaptive, and robust ML solutions in industrial composite processing.

### 6.1. Key Implications

For example, Ma et al.’s [102] image-recognition-driven FE workflow uncovered subtle wetting–evaporation couplings on complex porous surfaces, which would be difficult to capture with conventional continuum models alone. Likewise, the deep-material network in Wei et al.’s [111] LS-DYNA implementation delivered nonlinear anisotropic responses at multiscale fidelity in seconds—a task that direct numerical simulation (DNS) cannot match in any real-world timescale. These hybrid physics–ML frameworks not only accelerate design iterations but also open the door to real-time control and adaptive optimization.

Second, the breadth of sensor and vision applications—from ultrasonic phased array scans (Tunukovic et al. [114]) to hyperspectral surface imaging and synchronized X-ray laminography (Castro et al. [135])—reveals that ML can extract actionable insights from data streams too complex or noisy for heuristic processing. Supervised and unsupervised methods have both proven capable of identifying defects, monitoring cure fronts, and quantifying uncertainty with high reliability, reducing human inspection time from hours to seconds and improving detection metrics (e.g., >94% accuracy for leak detection and a 17% F1 score increase in ultrasonic inspection).

### 6.2. Limitations and Future Directions

However, several weaknesses and challenges temper these successes. Most frameworks rely on extensive high-fidelity training data—whether from expensive FE runs, synchrotron experiments, or exhaustive physical testing—raising questions about scalability and transferability across materials, geometries, or operating regimes. For instance, the Taguchi-plus-boosted-tree model for filament--wound cylinders (Azizian & Almeida [106]) must be retrained for each new winding pattern or resin system, limiting plug-and-play applicability in diverse manufacturing lines. Similarly, deep-RL agents for flow-media placement (Szarski & Chauhan [131]) typically require thousands of simulated episodes before generalizing to new laminate shapes, constraining their utility in fast-evolving production environments.

From an industrial perspective, the greatest promise lies in methods that integrate directly with existing process control architectures. Digital twins—such as Tannous et al.’s [125] reverse-flow SMC model or Stieber et al.’s T-RTM mold monitoring system—illustrate how ML can be embedded within plant-scale automation to guide material dosing, sensor placement, and quality assurance in real time. Yet, many academic proofs of concept stop short of demonstrating closed-loop feedback in a live production line. Issues of model robustness under sensor drift, data privacy in proprietary environments, and regulatory validation for safety-critical applications (e.g., aerospace hydrogen tanks or medical prostheses) remain open.

While the proposed hybrid AI framework demonstrates architectural integration of physics-based simulation, vision-guided inspection, and reinforcement learning-based control, its current validation relies solely on synthetic data. Although the dummy pipeline confirms that all modules interface correctly and produce outputs of the expected form, this approach does not capture the complexity, noise, and variability inherent in real NFRP fabrication. Consequently, the framework’s practical generalization to diverse fiber types and robustness under real-world process conditions remain untested. To address the reliance on synthetic data, the concept implemented the following: run a multi-scale validation campaign with synchronized multimodal data (HSI, RGB, T, force, XCT), calibrate sensors and model real noise/BRDF, apply transfer learning and domain adaptation to align sim and production domains, define clear acceptance metrics and robustness tests, perform hardware-in-the-loop validation with safe action bounds and uncertainty quantification, and deploy incrementally (≈50–200 lab runs → pilot → periodic retraining).

Looking forward, advancing industrial relevance will require three complementary efforts:

- Reducing reliance on large labeled datasets by leveraging physics-informed neural networks, meta-learning, or synthetic-to-real domain adaptation will broaden applicability to new materials and geometries without costly retraining.
- Developing modular, open-architecture twins that can ingest multisensor data, execute surrogate-accelerated simulations, and issue control recommendations will accelerate adoption across domains from automotive to wind-energy composites.
- Establishing benchmarks for ML model resilience to noise, missing data, and drift—as well as regulatory pathways for approving AI-driven inspection and design tools—will be essential for deployment in safety-critical industries.

In sum, the fusion of ML with composite manufacturing holds transformative potential for accelerating design cycles, improving quality, and reducing waste. Realizing this potential at scale, however, hinges on overcoming data and integration hurdles and on forging closer partnerships between ML researchers, process engineers, and industry stakeholders.

## 7. Conclusions

The integration of physics-based surrogates, advanced sensing, and machine learning is demonstrated to transform composite manufacturing. XPBD surrogates achieve approximately 100× speedup in draping simulations with <5% error, facilitating reinforcement-learning-based tension control for complex geometries. Hyperspectral imaging combined with U-Net segmentation classifies laser-structured FRP surfaces at >80% accuracy and reduces material waste by >30%. A CNN-based surrogate-based optimization framework predicts draping parameters in milliseconds with <5% FEM error across novel molds. Synchronized pultrusion sensing with real-time Kalman filtering (≤10 ms latency) establishes a foundation for digital twins. GBDT and ANN models estimate GFRP fracture toughness (<10% error) and pultruded column capacity (R>0.95), respectively.

Future research priorities include validation at industrial scale, development of uncertainty quantification methods, design of multi-modal sensor fusion architectures, adoption of graph neural network surrogates, investigation of sustainable materials, deployment of edge-AI models, and establishment of open benchmarks.

## Figures and Tables

**Table 1:** Comparative analysis of composite manufacturing processes.

Process	Challenges	Advantages	Core ML Requirements	Scientific Basis
Spray-up process	Control of resin flow and fiber alignment [46] Process variability due to material differences [47] Defect detection and correction [48]	Enhanced product quality [49] Reduced material waste [50] Real-time adjustments for optimal performance [51]	RL for roller path optimization [52] CNN-based defect detection [53] Resin spray control [54]	Fiber–resin deposition dynamics [15]; Roller contact mechanics [55]
Robotic Draping	Adapting to complex geometries [56] Maintaining fabric tension and alignment [57] Minimizing wrinkles and defects [58]	Increased flexibility in handling diverse geometries [56] Improved accuracy in fabric placement [59] Reduced labor costs [58,60]	XPBD surrogates for RL [61] Latent space geometry encoding [62] Sparse reward shaping for RL optimization [61]	Constraint mechanics $C_{s,ij},C_{b}$; Shear/bending energy minimization [59]
Pultrusion	Precise temperature and pull speed control [63] Resin cure kinetics management [64] Real-time defect detection [65]	Consistent product quality [66] High production rates [66] Improved mechanical properties of products [67]	Synchronized sensor fusion [65] Cure kinetics prediction models [68] Mechanical property surrogates [69]	Arrhenius viscosity η(T) [70]; Rule-of-mixtures $E_{L}$ [71]; Buckling criteria $P_{cr}$ [72]
Filament Winding	Fiber tension control [73] Uniform resin distribution [74] Path optimization for complex shapes [75]	Enhanced product strength and uniformity [76] Adaptability to various shapes and sizes [77] Reduced material wastage [78]	RL for path optimization [79,80] CNN for fiber alignment monitoring [81]	Mechanics of composite layup [82]; Resin flow dynamics [83]
Resin Transfer Molding (RTM)	Resin flow control and uniformity [84] Mold filling optimization [85] Minimizing void formation [86]	High-quality surface finish [87] Reduced cycle times [88] Enhanced structural integrity of products [89]	Predictive modeling for resin flow [90] Real-time monitoring and adjustments using sensors [91]	Fluid dynamics in porous media [90]; Thermoset resin curing kinetics [92]
Automated Fiber Placement (AFP)	Precision in fiber placement [93] Adaptability to part geometry [94] Real-time defect detection [95]	High accuracy and repeatability [96] Ability to handle complex geometries [78] Improved material utilization [97]	Computer vision for defect detection [98] Geometry-adaptive CNNs [99]	Mechanics of composite materials [100]; Fiber steering dynamics [101]

**Table 2:** Summary of recent ML-enabled methods in fiber-reinforced composites.

Authors	Year	Application	ML Method	Key Findings
Ma et al. [102]	2024	Wetting on porous tobacco surfaces	Image recognition + FE simulations	Predicted droplet spread and infiltration; linked microstructure to evaporation dynamics.
Li et al. [103]	2024	Online broken-filament detection in carbon fiber winding	RetinaNet w/ multiscale kernel fusion + strand-numbering	Enabled in-line detection and tracking of broken fibers with high accuracy.
Yao et al. [104]	2022	EMC selection for solder-joint reliability	Multi-fidelity Bayesian optimization + ANN	Identified optimal epoxy formulations minimizing creep strain under thermal cycling.
Dos Santos & Castro [123]	2025	Imperfection-sensitive cylinder design	SVM, Kriging, Random Forest surrogates	Mapped mass–stiffness trade-offs; RF achieved best inverse-design performance.
Perin et al. [105]	2023	Injection-mold gate optimization	Gradient boosting + FVM simulations	Predicted gate locations to improve local stiffness by up to 27%.
Castro et al. [135]	2021	In situ RTM impregnation monitoring	Segmentation network on SR-CL images	Quantified void formation and flow front evolution in woven preforms.
Azizian & Almeida [106]	2022	Uncertainty in filament--wound cylinders	Taguchi screening + boosted-tree regression	Predicted reliability under pressure; identified critical ply-thickness uncertainties.
Esmaeili et al. [107]	2022	Vacuum-bag leak detection	Electrical-circuit analogue + ML classifiers	Achieved >94% leak-location accuracy; slashed diagnosis time to seconds.
Ren et al. [110]	2024	Topological defect identification in nematics	Object detection + vision transformers	Automated recognition of ±1/2 defects with >90% accuracy across varied textures.
Wei et al. [111]	2023	Multiscale modeling of SFRC in LS-DYNA	Deep Material Network	Predicted nonlinear anisotropy at speeds orders of magnitude faster than DNS.
Tunukovic et al. [114]	2025	Ultrasonic PAUT analysis of CFRP	Multi-model AI pipeline (supervised, unsupervised, self-supervised)	Boosted F1 by 17.2%, reduced inspection time to <2 min.
Alshannaq [115,116]	2024/25	Pin-bearing strength of pultruded GFRP	Gradient boosting on the literature data	Revealed biases in design codes; proposed revised strength formulas.
Wang et al. [136]	2024	RTM process metamodeling	PixelRNN image-based metamodel	Achieved 97.3% resin-flow prediction accuracy at half the simulation cost.
Chai et al. [117]	2024	RTM filling pattern dataset	— (dataset release)	Provided benchmark dataset for ML process modeling in composite molding.
Lee & Sohn [118]	2024	Buckling prediction of filament--wound shells	RF, XGBoost, SVR, ANN, etc.	RF and XGBoost outperformed linear methods, achieving lowest prediction errors.
Ivan et al. [132]	2022	Fiber orientation in injection molding	GA-optimized RSC + ANN surrogate	Halved orientation-prediction error; improved modulus and strength forecasts by 43–59%.
Pfrommer et al. [122]	2018	Textile draping parameter optimization	Deep ANN surrogate in surrogate-based optimization	Reduced FE simulations needed and improved best-known draping solution.
Causon et al. [134]	2024	Real-time Bayesian inversion in RTM	Ensemble Kalman Inversion + ANN surrogate	Estimated local porosity/permeability in ≤ 1 s with confidence intervals.

**Table 3:** Comparative summary of ML approaches in composite manufacturing.

Method	Strengths	Limitations	Industrial Feasibility
XPBD surrogate for RL-driven draping (Blies et al.)	Fast simulation vs. FEM; stable constraint solving; experimentally validated	Lower accuracy than FEM; requires detailed material input; residual corrections needed	High, suitable for robotic lay-up lines
HSI-guided prediction for FRP coating (Gebauer et al.)	Links laser structuring, HSI, DL; closed-loop rework; ∼80% accuracy	Sensitive to spectral noise; limited training data; equipment cost	Medium, feasible in high-value aerospace/automotive lines
CNN surrogate for variable-geometry draping (Zimmerling et al.)	Generalizes across geometries; reduces FEM calls; low prediction error	Needs retraining for unusual shapes; reward design sensitive	Medium–High, promising for flexible textile forming
Digitized data framework for pultrusion (Helfrich et al.)	Standardized OPC UA + MQTT; synchronized datasets; real-time filtering	Prototype stage; bandwidth and latency issues; integration challenges	High, strong enabler for ML-based process optimization
ML prediction of GFRP fracture toughness (Karamov et al.)	Uses standard test data; avoids fracture toughness tests; interpretable models	Dependent on dataset diversity; microstructure not fully captured	High, easy adoption in QC and design workflows
ML-driven axial load prediction in pultruded GFRP columns (Kajendran et al.)	Combines mechanics with ML; ANN outperformed RSM; captures B/t, H/B, *H* effects	Limited dataset; splitting not well modeled; no post-peak prediction	Medium–High, useful for design but needs larger datasets

## Data Availability

The code implementing the described experiments is available in the repository at XPBD-GNN (https://github.com/catauggie/XPBD-GNN, accessed on 7 August 2025). Researchers and enthusiasts can access, review, and contribute to the codebase for further exploration and collaboration.
